# The Role of Furin and Its Therapeutic Potential in Cardiovascular Disease Risk

**DOI:** 10.3390/ijms25179237

**Published:** 2024-08-26

**Authors:** Hannah Fry, Mohsen Mazidi, Christiana Kartsonaki, Robert Clarke, Robin G. Walters, Zhengming Chen, Iona Y. Millwood

**Affiliations:** Nuffield Department of Population Health, University of Oxford, Oxford OX3 7LF, UK; hannah.fry@ndph.ox.ac.uk (H.F.); mohsen.mazidi@ndph.ox.ac.uk (M.M.); christiana.kartsonaki@ndph.ox.ac.uk (C.K.); robert.clarke@ndph.ox.ac.uk (R.C.); robin.walters@ndph.ox.ac.uk (R.G.W.); zhengming.chen@ndph.ox.ac.uk (Z.C.)

**Keywords:** furin, cardiovascular disease, proteomics, genetics, drug targets

## Abstract

Furin is an important proteolytic enzyme, converting several proteins from inactive precursors to their active forms. Recently, proteo-genomic analyses in European and East Asian populations suggested a causal association of furin with ischaemic heart disease, and there is growing interest in its role in cardiovascular disease (CVD) aetiology. In this narrative review, we present a critical appraisal of evidence from population studies to assess furin’s role in CVD risk and potential as a drug target for CVD. Whilst most observational studies report positive associations between furin expression and CVD risk, some studies report opposing effects, which may reflect the complex biological roles of furin and its substrates. Genetic variation in *FURIN* is also associated with CVD and its risk factors. We found no evidence of current clinical development of furin as a drug target for CVD, although several phase 1 and 2 clinical trials of furin inhibitors as a type of cancer immunotherapy have been completed. The growing field of proteo-genomics in large-scale population studies may inform the future development of furin and other potential drug targets to improve the treatment and prevention of CVD.

## 1. Introduction

Despite improvements in treatment and prevention, cardiovascular disease (CVD) remains the most important cause of death and disability worldwide, with large variations in mortality rates between populations. The recent Global Burden of Disease (GBD) study reported that age-standardised CVD mortality rates varied six-fold between 21 geographic regions, with ischaemic heart disease (IHD) being the leading cause of disability-adjusted life years (DALYs) [1]. Hence, there is substantial unmet need for further improvements in preventative and treatment strategies to reduce the global burden of CVD, including the identification of novel targets and the repurposing of existing drug targets. The advent of high-throughput proteomic technologies and their application, together with genomic data, in large-scale population and clinical studies affords unique opportunities to identify clinically relevant biomarkers that could improve prediction of CVD risk [2] and inform drug development [3]. For instance, recent large-scale proteo-genomic analysis of East Asian and European ancestry populations identified 13 potential novel drug target proteins for IHD [4]. This study reported a potential causal association between higher plasma levels of furin and higher risk of IHD, raising the possibility that furin might represent a target for CVD prevention and/or treatment [4].

**Figure 1:**
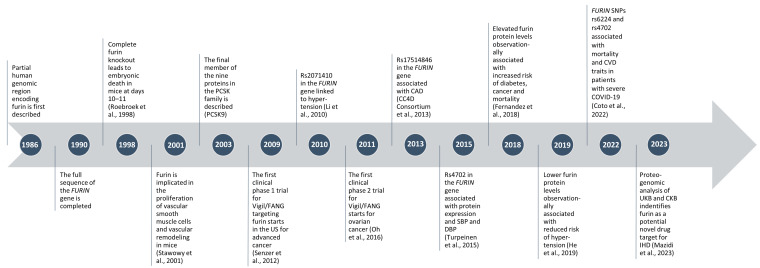
Key milestones since the discovery of furin including notable findings from epidemiological and genetic studies [4,11,14,19,20,21,22,23,24,25,26]. Abbreviations: PCSK = proprotein convertase subtilisin/kexin; Vigil/FANG = an immunotherapy designed to target furin expression; CAD = coronary artery disease; SBP = systolic blood pressure; DBP = diastolic blood pressure; CVD = cardiovascular diseases; UKB = UK BioBank; CKB = China Kadoorie BioBank; IHD = ischaemic heart disease.

First discovered in 1990, furin was the first of nine subtilisin-like proprotein convertases (PCSKs) to be identified, and extensive research conducted over the last three decades has highlighted its physiological importance and potential role in a range of cardiovascular phenotypes (Figure 1). Furin is encoded by the *FURIN* gene (previously named *PCSK3*) located on chromosome 15 and is a type 1 membrane-bound protease comprising 794 amino acids. Furin is ubiquitously expressed with relatively low tissue specificity [5]. According to the Adult Genotype Tissue Expression (GTEx) database, expression is highest in the liver, while whole blood, the aortic artery, and the coronary artery have the fourth, sixteenth, and nineteenth highest levels of expression, respectively (in terms of median transcripts per million) out of 54 tissue types covered (data source: GTEx Analysis Release V8 [dbGaP Accession phs000424.v8.p2]; https://www.gtexportal.org/home/gene/FURIN#geneExpression; accessed on 4 July 2024). Within the cell, furin is localised to the Golgi apparatus and the nucleoplasm, where it plays a key role in the conversion of proteins and hormones from their inactive precursors (known as proproteins or prohormones) to their active forms by proteolytic cleavage at specific amino acid residues, a critical step in many protein pathways related to homeostasis, embryogenesis, and even viral pathogenesis [6,7,8,9]. The latter became particularly evident during the recent SARS-CoV-2 pandemic due to a furin-specific cleavage site in the spike protein of the virus [10], and several associations were reported between *FURIN* expression and cardiovascular complications or comorbidities in infected individuals [11,12,13]. The importance of furin in cardiac development was reported as early as the 1990s, when Roebroek et al. discovered that complete furin knockdown in mice resulted in embryonic death at day 10–11 due to cardiac tissue malformation [14]. It has since been reported that furin processes upwards of 400 substrates [8], including many proteins and hormones related to cardiac function, and therefore may influence CVD risk via its action on these physiological pathways [15]. Some of these substrates include transforming growth factor β1 (TGF-β1), implicated in myocardial fibrosis [16], pro-brain natriuretic peptide (proBNP), a hormone expressed in the cardiac tissue and an established biomarker for certain CVD phenotypes [17], and PCSK9, implicated in lipid regulation [18]. However, due to its ubiquitous expression patterns and complex mechanisms of action, the advantages of pursuing furin as a drug target for CVD, and any possible adverse effects, remain to be fully elucidated [15].

Here, we undertake a critical assessment of observational and genetic population studies of furin in relation to CVD and present our findings in a narrative review. Our search used the PubMed (https://pubmed.ncbi.nlm.nih.gov/ accessed on 15 January 2024) and GWAS Catalog (https://www.ebi.ac.uk/gwas/ accessed on 15 January 2024) online databases to identify studies which associated furin with CVD risk factors and outcomes, and also identified clinical studies of treatments targeting furin (https://clinicaltrials.gov accessed on 15 January 2024). Human studies using cross-sectional, interventional, case-control, or prospective designs are included, and several in vitro, in vivo, and animal studies are described. Studies looking at SARS-CoV-2 infection and comorbidities were largely excluded, as this was not a focus for the current review. Our main objectives were (1) to evaluate the available evidence linking furin with CVD risk factors and outcomes in population studies; (2) to identify clinical studies of drug treatments targeting furin; and (3) to discuss research approaches to further understand the relevance of furin for the treatment and prevention of CVD.

## 2. Risk Factors for Cardiovascular Diseases

### 2.1. Blood Pressure Traits

Elevated blood pressure accounted for >10 million CVD deaths worldwide in 2021 and is one of the most important modifiable risk factors for CVD [27]. It has previously been reported that furin may be involved in blood pressure regulation via the renin–angiotensin system and sodium–electrolyte balance through cleavage of the pro-renin receptor (PRR) and activation of epithelial sodium channels (ENaC) [19,28], and there is growing evidence from genetic studies of a role of *FURIN* in blood pressure phenotypes. In an early genetic study of 1000 participants of Kazakh and Uyghur Chinese ancestry, the G allele of rs2071410 in *FURIN* was identified as a possible independent risk factor for hypertension in this population [19] (Table 1). Similar results were also reported by Ganesh et al., who demonstrated significant associations between the same single nucleotide polymorphism (SNP) and diastolic blood pressure (DBP) in 61,619 Europeans, and between rs6227 and both systolic blood pressure (SBP) and mean arterial pressure (MAP) [29]. Genome-wide analysis of 69,395 Europeans and 29,719 East Asians as part of the International Consortium for Blood Pressure (ICBP) identified a significant association between rs2521501 in *FES-FURIN* and both SBP and DBP, but not hypertension [30]. The *FES* gene is located close to *FURIN* on chromosome 15 (*FURIN*; *FES* upstream region). Further analysis by ICBP demonstrated an association between another *FURIN* SNP––rs8032315––and SBP, but not DBP [31]. Associations between different *FURIN* variants and blood pressure measures have been confirmed in multiple large-scale genetic studies [32,33,34,35,36,37,38,39,40,41] (Table 1). Additional associations with other blood pressure-related phenotypes have also been reported. Kiiskinen et al. found that rs8032315 was associated with the use of blood pressure-lowering medication in GWAS studies in three European cohorts [42], and associations have been identified between rs6224 and preeclampsia and gestational hypertension [43,44], in addition to potential ancestry-dependent associations of four *FURIN* SNPs with post-exercise hypotension in hypertensive individuals in the USA [28] (Table 1). The latter study highlights the need for further large-scale multi-ancestry prospective studies to further clarify possible ancestry-dependent genetic effects.

A small number of studies have investigated the associations of plasma levels of furin with blood pressure. Research conducted in 1428 individuals from the Young Finns Study (YFS; a prospective cohort in Finland) identified the cis-expression quantitative trait locus (cis-eQTL) rs4702, which was significantly associated with blood pressure [20]. Interestingly, the rs4702 AA genotype was associated with lower levels of furin mRNA and higher SBP and DBP after adjusting for age and sex, although *FURIN* expression was upregulated in the peripheral blood cells in participants with higher levels of blood pressure [20]. Similarly, in a combined cross-sectional and prospective analysis of serum furin and hypertension in 2312 elderly Chinese adults, lower plasma furin levels were associated with higher levels of SBP, DBP, and MAP, in addition to higher risks of hypertension during the follow-up period [21] (Table 2). Moreover, analysis of expression data from three small datasets on hypertensive individuals in the Gene Expression Omnibus (GEO) database identified *FURIN* as one of 12 hub genes related to hypertension and reported an inverse correlation of *FURIN* expression with hypertension, suggestive of an inhibitory role of *FURIN* in the development of hypertension [47]. Further, Mazidi et al., found an association between plasma furin protein levels and SBP in an observational analysis of 1463 proteins in Chinese adults [4]. Consequently, there is a need for additional well-designed prospective studies of the associations of furin with blood pressure and hypertension.

### 2.2. Blood Lipids

Low-density lipoprotein cholesterol (LDL-C) is a leading modifiable risk factor for CVD, accounting for >3.5 million CVD deaths worldwide each year [27]. Furin may play an important role in lipid metabolism, as seen in hepatic tissue studies in mice which demonstrate its role in the cleavage of endothelial lipase and lipoprotein lipase, which are important in the regulation of high-density lipoprotein cholesterol (HDL-C) and cholesterol homeostasis [48,49]. Likewise, there is evidence of an association between furin and blood lipids from several human population-based observational studies. For example, Fernandez et al. reported significant associations between plasma furin levels and both LDL-C and HDL-C concentrations in a cross-sectional analysis of 4678 individuals in Sweden [22], and Wang et al. reported that individuals in the highest tertile of furin concentration were more likely to have higher LDL-C levels [50].

**Table 2:** Summary of associations between furin protein levels or activity and cardiovascular risk factors.

Study Design	Population	Primary Endpoints	Protein Measurement	Key Findings	Reference
Blood pressure traits:					
Cross-sectional and prospective (Suzhou, China)	Middle-aged and elderly residents in the community (N = 2312)	Hypertension, SBP, DBP, MAP	Furin serum levels measured using ELISA	Cross-sectional: lowest quartile of furin concentration compared to the highest had higher SBP, DBP, and MAP Prospective: those in the lowest quartile of furin compared to the highest had a higher risk of hypertension	[21]
Prospective (Malmö, Sweden)	Residents aged 45 to 69 years (N = 4678)	Diabetes, CAD, all-cause mortality, cause-specific mortality	Furin serum levels measured using PEA (Olink)	Baseline furin concentration strongly associated with SBP, DBP, and antihypertensive treatment	[22]
Blood lipids:					
Prospective (Malmö, Sweden)	Residents aged 45 to 69 years (N = 4678)	Diabetes, CAD, all-cause mortality, cause-specific mortality	Furin serum levels measured using PEA (Olink)	Baseline furin concentration strongly associated LDL and HDL	[22]
Nested case-control (Västerbotten, Sweden)	Residents aged > 40 years (N = 276)	MetS (based on score developed using BMI, triglyceride levels, total-C levels, mid-blood pressure, and fasting glucose levels) and CRC	Furin serum levels measured using PEA (Olink)	Furin levels positively associated with MetS score, BMI, and triglyceride levels (Bonferroni-adjusted *p* < 0.5)	[51]
Cross-sectional (Suita, Japan)	CAD patients with HeFH (N = 138)	Intravascular ultrasound measures (PAV, vessel volume, imaged length, and lumen volume)	Total, mature, and furin-cleaved PCSK9 (fc-PCSK9) measured using ELISA	No significant association between fc-PCSK9 and PAV, vessel volume, or lumen volume Fc-PCSK9 was not a significant predictor of PAV	[52]
Prospective, (Suita, Japan)	City residents aged 30–79 years (N = 1436)	CAD (including AMI, sudden cardiac death, and stable CAD)	Total, mature, and fc-PCSK9 measured using ELISA	Fc-PCSK9 was positively associated with SBP, BMI, high-sensitivity CRP levels, and DBP at baseline	[53]
Randomised clinical trial (Iwate, Japan)	STEMI patients (N = 36)	Serum mature and fc-PCSK9 and serum Lp(a)	Mature and fc-PCSK9 measured using ELISA	Fc-PCSK9 levels were significantly reduced with administration of evolocumab	[54]
Diabetes and adiposity:					
Prospective (Malmö, Sweden)	Residents aged 45 to 69 years (N = 4678)	Diabetes, CAD, all-cause mortality, cause-specific mortality	Furin serum levels measured using PEA (Olink)	Baseline furin concentration strongly associated with BMI, glucose, insulin After adjusting for conventional risk factors, increase in furin concentration is associated with higher risk of diabetes Higher furin concentration associated with lower survival rate	[22]
Prospective (China)	Middle-aged and elderly residents in the community in Suzhou (N = 892)	Abdominal obesity (WC ≥ 85 cm for males and 80 cm for females)	Furin serum levels measured using ELISA	Lower furin serum levels were associated with higher BMI, WC, and prevalent diabetes at baseline Individuals in the lowest tertile of serum furin had a higher risk of developing abdominal obesity compared to those with highest tertile	[55]
Case-control (Gaza Strip, Palestine)	Hospital patients referred to diabetic clinic, hospital cardio care unit and healthy subjects from routine check-ups (N = 75)	T2D with and without cardiovascular comorbidities	Furin serum levels measured using ELISA	Significantly higher furin concentration in T2D patients with CVDs than those without and healthy controls Furin had a high sensitivity (80%) and specificity (96%) for diagnosing CVDs in T2D patients	[56]
Cross-sectional (Suzhou, China)	Middle-aged and elderly residents in the community (N = 2172)	Diabetes (>7.0 mmol/L) and prediabetes (5.6–6.9 mmol/L)	Furin serum levels measured using ELISA	Lower log-furin levels associated with higher levels of fasting plasma glucose Lower log-furin levels borderline associated with increased risk of diabetes and prediabetes	[57]

Search strategy: studies were identified using PubMed (https://pubmed.ncbi.nlm.nih.gov/ accessed on 15 January 2024; search terms: “Furin, CVD”, “Furin, cardiovascular disease”), and abstracts were screened manually for mention of any CVD-related outcomes or risk factors, including IHD, stroke, coronary artery disease (CAD), myocardial infarction (MI), blood pressure, hypertension, blood lipids, diabetes, and heart failure (HF). Abbreviations: SBP = systolic blood pressure; DBP = diastolic blood pressure; MAP = mean arterial pressure; ELISA = enzyme-linked immunosorbent assay; CAD = coronary artery disease; T2D = type 2 diabetes; CVDs = cardiovascular diseases; MetS = metabolic syndrome; BMI = body mass index; total-C = total cholesterol; PAV = percent atheroma volume; CRC = colorectal cancer; PEA = proximity extension assay; STEMI = ST segment elevation myocardial infarction; AMI = acute myocardial infarction; WC = waist circumference; CRP = c-reactive protein; HeFH = heterozygous familial hypercholesterolemia; PCSK9 = proprotein convertase subtilisin/kexin type 9; Lp(a) = lipoprotein a.

Findings from genetic studies show associations between two SNPs in *FURIN* that also associated with higher expression levels (rs6224 and rs4702) and increased risk of hypercholesterolemia in COVID-19 patients admitted to intensive care units (ICUs) [11]. Moreover, associations have also been reported between rs8039305 and the use of HMG CoA reductase inhibitors (also known as statins) used to treat hyperlipidaemia [33].

Metabolic syndrome (MetS) is defined as a combination of obesity, high blood pressure, insulin resistance, and high LDL-C and triglycerides, and is known to associate with higher risks of type 2 diabetes (T2D) and CVD. Findings from a GWAS of 2988 individuals demonstrated an association of rs17514846 in the *FURIN* gene with MetS in a case-control study of Japanese adults, after adjustment for age, sex, and smoking status [46]. Interestingly, the authors found that the A allele of rs17514846 may have a protective association with MetS, despite being previously reported as a risk allele for coronary artery disease (CAD) in a large, multi-ancestry GWAS [23]. A nested case-control study in Japanese adults found no significant association between rs17514846 and hypertriglyceridemia (triglycerides > 1.65 mmol/L) after adjusting for age, sex, and body mass index (BMI), but did report a borderline significant association between the A allele and reduced serum triglyceride levels [45]. Although neither study included plasma furin levels as part of their investigations, the A allele in this SNP has been reported to associate with increased *FURIN* expression and protein levels [58,59,60,61]. It would therefore be interesting for studies investigating the association between *FURIN* variants and MetS risk to also assess protein levels. One study that did include furin measurements in their analysis of 276 adults in Sweden found that plasma protein levels were positively associated with metabolic syndrome score (based on multiple adiposity measures and blood lipid and glucose levels) [51] (Table 2). These apparently contrasting results may reflect differences in study designs, study populations, and the definition of MetS used. For example, some used a clinical diagnosis of MetS, whilst the other used a derived score based on several different measures. It could also reflect levels of residual confounding, or perhaps raises the question of the physiological importance of circulating levels of plasma furin. Furin protein cycles between the trans-Golgi network and cell surface membrane, proteolytically cleaving its substrates endocellularly [6]; therefore, plasma levels may relate to cell damage or other physiological changes that may affect risk of, or be a consequence of, CVD.

Another possible mechanism through which furin may regulate lipid metabolism is its interaction with PCSK9. PCSK9 belongs to the same family of proprotein convertases as furin and has been studied extensively due to its role in regulating LDL-C. LDL-C is cleared from the circulation by low-density lipoprotein receptors (LDLRs), and degradation of these receptor proteins by PCSK9 results in elevated levels of LDL-C in the plasma [62,63]. However, PCSK9 can present as different subtypes which have different biological roles. This includes a mature form and a furin-cleaved form (fc-PCSK9). PCSK9 is produced in the liver and undergoes autocatalytic cleavage to become the mature form, which can then undergo subsequent cleavage by furin to form the smaller fc-PCSK9 subtype [64]. Fc-PCSK9 is thought to have around 50% lower ability to metabolise LDLRs than mature PCSK9, and has a shorter half-life [64]. This suggests a potential protective effect of furin on CVD, as a higher proportion of mature PCSK9 could result in more efficient degradation of LDLR and hence higher levels of inflammatory markers and lipids which promote atherogenesis [52]. A study of 138 individuals with heterozygous familial hypercholesterolemia (HeFH) and CAD found significantly higher levels of mature PCSK9 than fc-PCSK9, and fc-PCSK9 was not associated with any intravascular ultrasound measures (including percent atheroma volume, blood vessel volume, and lumen volume) with adjustment for age, sex, other clinical characteristics, and blood biomarkers [52] (Table 2). This would seem to support the theory that the mature rather than the furin-cleaved subtype of PCSK9 may have greater importance in CVD risk and progression. However, there is evidence that fc-PCSK9 may be independently associated with a higher risk of CAD events and stroke [53]. In a prospective cohort study of 1400 Japanese adults followed up over 13 years, fc-PCSK9 level was a predictor of future coronary events and was positively associated with BMI and high-sensitivity C-reactive protein (CRP), a known biomarker for inflammation [53]. These findings suggest that fc-PCSK9 may also be important in the progression of atherosclerosis and the development of CVD, and therefore any targeted inhibition of furin could potentially help prevent CVD by additionally reducing levels of fc-PCSK9. Indeed, one small, open-label, 1:1 randomised trial involving 36 participants reported that fc-PCSK9 levels were transiently upregulated following myocardial infarction (MI) and that these levels were significantly reduced with treatment of the PCKS9 inhibitor evolocumab [54]. Separate findings from another study of 126 patients with ST-elevation myocardial infarction (STEMI) demonstrated the prognostic relevance of the ratio of circulating fc-PCSK9 to mature-PCSK9 48 h after a percutaneous intervention procedure [65]. Although this study did not find any association between fc-PCSK9 and major adverse cardiovascular events (MACEs), as Kataoka et al. reported, they did find a similar association with CRP, providing further support for the role of fc-PCSK9 in atherosclerosis progression via an inflammatory pathway.

Some furin inhibitors have demonstrated effectiveness in reducing furin activity or expression when used in lipid metabolism model studies. For example, inhibition of furin with profurin has been shown to decrease plasma LDL-C levels and slow atherosclerotic progression in mice [66], as well as reduce plasma levels of phospholipid transfer protein (PLTP) [67]. Profurin acts as a natural inhibitor to furin in its mature state [68], and these findings warrant replication in further studies targeting furin activity in lipid regulation pathways. Another synthetic inhibitor, decanoyl-RVKR-CMK, has been demonstrated to reduce furin’s ability to cleave PCSK9, resulting in levels of mature PCSK9 four times higher than fc-PCSK9 in treated media [64], which may be relevant for therapies aimed at reducing levels of different subtypes of PCSK9. Additionally, furin inhibition by decanoyl-RVKR-CMK reduces the migratory ability and proliferation of monocytes, which play a key role in atherosclerotic progression [69]. These small molecules may be possible options for further consideration of furin inhibition as a form of therapy for lipid- or inflammation-related CVD.

### 2.3. Diabetes and Adiposity

The GBD study reported that an estimated 1.95 and 2.30 million global CVD deaths were attributable to elevated BMI and fasting plasma glucose levels, respectively, in 2021 [27]. These phenotypes are important in the progression of diabetes and subsequent risk of CVD. Previous evidence from animal studies has been supportive of a positive association between furin expression and obesity; one study reported upregulated furin expression in the inflamed adipose tissues of obese mice, linking furin to obesity-related inflammation and potentially CVD [70]. However, observational findings from human studies have not been universally concordant. He et al. reported that elderly Chinese adults in the lowest tertile of plasma furin concentrations had a higher risk of abdominal obesity compared to those in the highest tertile over a follow-up period of 4 years, and that lower furin serum levels were positively associated with prevalent diabetes [55]. However, findings from a case-control study of 50 T2D cases with (n = 25) and without (n = 25) CVD in Gaza reported higher plasma levels of furin in T2D cases with CVD compared to both T2D cases without CVD and healthy controls [56] (Table 2). The latter study reported that furin levels had a high sensitivity and specificity of detecting CVD in patients with T2D and had higher positive predictive value, greater diagnostic accuracy, and area under the curve than BNP (another indicative biomarker for CVD) [56]. A prospective study of 4678 participants in Sweden with a median follow-up period of 21.3 years also reported that higher plasma levels of furin were independently associated with higher risks of incident diabetes after adjusting for other CVD risk factors and demographic confounders [22]. Additional analysis in the same Chinese cohort as the former study found that log-transformed serum furin levels were similarly inversely associated with fasting glucose levels and risk of prediabetes and diabetes, and when divided into quartiles of furin concentration, those in the lowest quartile were at higher risk compared to those in the highest quartile (prediabetes: OR = 1.42, 95% CI = 1.05–1.92, *p* = 0.023; diabetes: OR = 1.80, 95% CI = 1.13–2.91, *p* = 0.015) [57]. Other observational studies such as Wang et al. alternatively support an association between higher furin levels and diabetes risk and have previously reported an association between the highest tertile of plasma furin concentration and prevalent diabetes in their study of >1000 participants in China [50]. Conflicting results from these cross-sectional findings in East Asian populations may reflect regional differences or differences in reverse causation or adjustment for covariates in the statistical analysis, suggesting a need for further large well-designed studies to elucidate the relationships between furin levels and adiposity and metabolic traits.

## 3. Cardiovascular Diseases

### 3.1. Coronary Artery Disease (CAD)

Atherosclerosis is a key risk factor for progression of CAD, and there are a number of in vitro and in vivo studies that provide evidence of the possible mechanism through which furin might play a role in atherosclerotic progression and CAD risk. Elevated furin expression levels have been demonstrated in macrophages in atherosclerotic lesions and have been implicated in the activation of matrix metalloproteinases (MMPs), with the related proteolytic cascade potentially increasing the risk of atherosclerosis [71]. Additionally, furin expression has been found to be upregulated in vascular smooth muscle cells in rats after arterial injury, and has been implicated in their proliferation and in vascular remodelling [24]. Overexpression of furin in isogenic cell lines has also been demonstrated to lead to an increase in the migratory ability and proliferation rates of macrophages and monocytes, which play a central role in inflammation, atherosclerosis development, and later CAD risk [58]. Moreover, analysis of cultured vascular endothelial cells showed that the A allele of rs17514846 in *FURIN* is associated with a higher expression of furin and that *FURIN* knockdown inhibited monocytes’ adhesion and reduced their migratory ability [59]. Immunohistochemistry and PCR conducted on atherosclerotic plaques taken from participants in the Tampere Vascular study in Finland showed that furin was the highest and most consistently overexpressed member of the PCSK family of proteins [72]. However, whilst these findings suggest that there might be a positive association of furin expression with atherosclerotic progression, other studies suggest that furin may inhibit this pathway by regulating the lipid content in macrophages and promoting autophagy [73]. Thus, the available evidence for furin in atherosclerosis from molecular studies is not wholly consistent. Evidence from population studies on protein abundance and atherosclerotic progression is more consistent and tends to lend more support to a positive association between higher furin concentrations and increased levels of relevant atherosclerotic markers. Analysis of the Bruneck study, a population-based study of 826 participants in Italy, reported that carriers of the A allele of rs17514846 had higher levels of circulating monocyte chemotactic protein-1 (MCP-1; a known inflammatory marker) and greater coronary intima media thickness (CIMT; an important indicator of carotid atherosclerosis) [59]. Moreover, higher plasma levels of furin were found in deceased CAD cases with lower histological evidence of coronary atherosclerosis, suggesting that furin activity may have greater importance in the earlier stages of atherosclerosis progression [60]. Yang et al. offer some additional explanation of the mechanism through which furin may act to promote CAD progression by reporting that higher expression in isogenic cell lines with the rs17514846 A/A genotype compared to C/C could be related to methylation of the C allele, which allows binding of transcription factor methyl-CpG binding protein 2 (MeCP2; responsible for repressing other protein-coding genes), thereby regulating *FURIN* expression [69].

This evidence from molecular studies has been elaborated upon by several large-scale genetic studies. Associations between SNPs in *FURIN* and CAD have been widely reported, and include rs17514846 [23,39,74], rs8032315 [31,42,75], rs8027450 [75], rs4932371 [76], and rs4932373 [77] in *FES* (Table 3). Rs17514846 was associated with higher levels of furin in myocardial tissues of deceased patients with CAD, although this SNP was unrelated to the severity of coronary atherosclerosis [60]. Interestingly, the same study did find that levels of both coronary arterial and myocardial furin levels were associated with atherosclerotic severity [60]. They also found that the AA genotype of rs17514846 was associated with elevated myocardial protein levels but was not associated with coronary levels [60]. This is supported by several other studies that have identified associations between this SNP and furin expression [58,59,61]. Furthermore, pathway and gene-set enrichment analysis of 9889 cases and 11,089 controls in seven CAD GWAS datasets identified *FURIN* as likely to be a critical component in several pathways in atherosclerosis and CAD development [78].

GWAS analysis of CAD in the UK Biobank (UKB) suggested that *FES* might be the putative causal gene rather than *FURIN*, and found that the same genetic signal was associated with higher furin expression in the blood and lower risk of CAD [61]. The authors suggested that *FES* may regulate *FURIN* expression and that further studies should investigate the role of *FES* in CAD to elucidate the underlying relationship [61]. Future genetic studies on these loci and their associations with CAD risk could provide further evidence on directions of effect in relation to gene expression, protein levels, and CAD risk.

Recently, evidence reported by Mazidi et al. from a nested case-cohort study of 3977 participants including 1976 IHD cases in the China Kadoorie Biobank (CKB) is supportive of an association between plasma furin protein levels and increased risk of incident IHD (HR = 1.236, SE = 0.062, FDR = 0.005 per SD increase in protein concentration) [4]. In addition, two-sample Mendelian randomisation involving data from the UKB and the CARDIoGRAM*plus*C4D (CC4D) consortium showed that a genetic variant (rs1029420) in *FURIN* had a potential causal association with IHD in European ancestry populations (OR = 1.32, 95% CI = 1.26–1.39 per SD increase in protein concentration) [4]. There is also evidence that furin protein levels may play an important role in prognosis following a CAD event. For example, an observational analysis of 1100 acute myocardial infarction (AMI) patients in China reported a significant association of elevated plasma furin levels with risk of non-fatal MI, but not with MACE or cardiovascular death [82] (Table 4). In contrast, prospective analysis of 1312 patients with STEMI and non-ST segment elevation myocardial infarction (NSTEMI) in China suggested that higher plasma levels of furin were significantly associated with MACE [50]. After adjusting for age, sex, clinical risk factors, and other biomarkers, participants in the highest tertile of plasma furin concentrations had a higher risk of all-cause mortality, MACE, cardiovascular death, recurrent MI, and rehospitalisation for heart failure (HF) compared to those in the lowest tertile [50]. Moreover, the addition of furin significantly improved the fit of risk prediction models developed using other well-established biomarkers [50]. Different findings between studies may be due to population differences, levels of adjustment for confounding, and case definitions and sample sizes (Table 4), and it is possible that pharmacotherapy use for CAD may have an impact on the variable results.

Associations with other phenotypes related to CAD have also been reported. For example, Sakaue et al. found an association between rs57515981 in *FURIN* and the risk of unstable angina pectoris in a large-scale GWAS of 220 phenotypes in BBJ, FinnGEN, and UKB participants [33] (Table 3). Additionally, Fernandez et al. reported an observational association between furin protein levels and death from CVD causes in minimally adjusted models; however, these findings were attenuated after adjustment for confounders [22].

Some small molecules known to inhibit furin activity have been used in cell line and animal studies to target areas of the pathways leading to atherosclerosis progression and increased CAD risk. Use of the furin inhibitor α1-antitrypsin Portland (α-1-PDX) slowed progression of the more severe atherosclerotic lesions and reduced overall plaque complexity in mice, reduced intimal thickness and total plaque area, and significantly increased HDL-C levels [85]. Moreover, it reduced expression of vascular cell adhesion molecule-1 (VCAM-1), chemokines, and cytokines in monocytes, macrophages, and vascular endothelial cells, in addition to lowering plasma levels of tumour necrosis factor alpha (TNF-α), interleukin-1 beta (IL1-β), and TGF-β1, highlighting the role of inflammation in mediating the effects of furin on risk of CVD [85]. Additionally, Zhao et al. successfully used short-hairpin RNAs (shRNAs) to inhibit furin by targeting certain regions of the transcript in isogenic monocyte cells, resulting in reduced migration and proliferation of macrophages and monocytes and increased apoptosis [58]. This evidence suggests that α-1-PDX and shRNAs are two more possible candidates for furin inhibition in prevention or treatment of atherosclerosis and warrant future investigations in development of therapies designed to reduce CAD risk.

### 3.2. Stroke

Micro-RNAs (miRNAs) are involved in the regulation of gene transcription and expression by targeting messenger RNA (mRNA) and play a role in protein regulation [86]. Recently, case-control analysis of data on middle cerebral artery occlusion (MCAO) in rats from the GEO database was used to identify differentially expressed mRNAs and miRNAs and found downregulation of *FURIN* in cases, which suggests that the miRNA molecule miR107-5p may promote stroke progression by inhibiting *FURIN* expression [87]. Despite a relatively small sample size, these findings from animal models are important for understanding the possible mechanisms through which furin may affect stroke risk and possible routes of targeted treatment via miRNAs. Evidence of an association between the *FURIN* gene and stroke has also been demonstrated in population-based studies, with a recent multi-ancestry GWAS meta-analysis identifying *FURIN* as a putative causal gene [81]. Notably, rs1573644 in *FURIN* was most significantly associated with large-artery atherosclerotic stroke (LAS), with a positive effect observed between the C allele of this variant and LAS risk [81]. Other studies report an increased risk of transient ischaemic attack (TIA) and poorer prognosis associated with the G allele of rs2071410 in *FURIN* amongst 753 East Asians [79], while rs4932370 was associated with increased odds of any ischaemic stroke in a trans-ancestral GWAS meta-analysis [80].

### 3.3. Other Cardiovascular Diseases

Elevated furin levels have been identified in rats following induced thoracic aortic aneurysms (TAAs) [88]. These levels were found to be regulated by miR-133a (another miRNA molecule that directly targets furin translation), whereby overexpression of this microRNA suppressed furin expression and attenuated progression of TAA, possibly through the latter’s role in activation of MMPs involved with tissue remodelling [88]. The role of another microRNA, miR-22-3p, has also been identified in regulating furin translation in rats with HF [89]. Silencing this molecule resulted in increased furin expression and improved cardiac function, suggestive of a protective role of furin in this case [89]. These studies suggest that furin levels are regulated by miRNAs and that different molecules may play different roles in the progression of CVD risk via this mechanism.

Evidence from animal models has also been useful for further understanding the relationship between protein levels of furin and other cardiovascular outcomes, as well as patterns of expression following physiological changes in cardiac tissues. For example, congestive heart failure (CHF) was associated with significantly higher levels of furin expression in rat models compared with sham controls, with higher levels of expression in pulmonary and cardiac tissues than in kidneys [90]. Moreover, artificially inducing HF in canines resulted in a significant increase in furin mRNA and protein expression in left atrial tissue compared to animals with normal cardiac function [91]. Additionally, it has previously been shown that protein levels of furin were significantly elevated in cardiac tissue following induced MI and HF in rats [16].

In humans, cross-sectional analysis of 683 acute decompensated heart failure (ADHF) and CHF patients reported no significant differences in plasma concentrations of furin between each case type; however, higher furin activity was observed in those with ADHF [83]. A case-control study of 63 patients with dilated cardiomyopathy (DCM) and ischaemic cardiomyopathy (ICM) found no significant difference in furin levels between the cases (combined or each separately) and the controls [84]. However, when ICM patients were compared to DCM patients, furin protein levels were significantly higher in the former [84]. Both studies were limited by sample size, which may have impacted their ability to detect an association if it was present between protein levels and the outcomes considered. Future studies could consider increasing the sample size and incorporating genetic data in order to make causal inferences on the role of furin and HF and cardiomyopathy subtypes.

## 4. Summary and Future Perspectives

**Figure 2:**
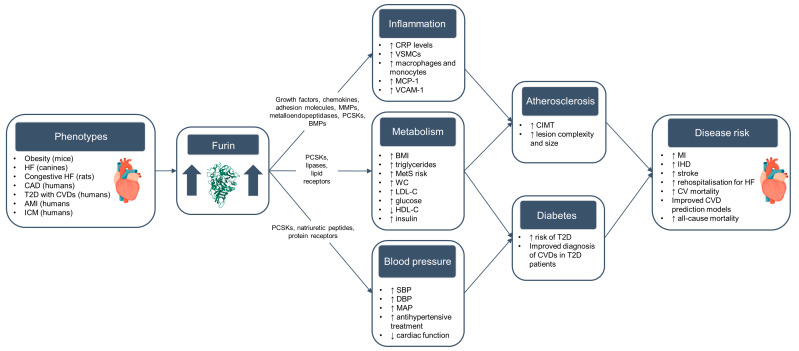
Associations between furin protein levels and risk factors and outcomes in key pathways related to cardiovascular disease *. Abbreviations: HF = heart failure; CAD = coronary artery disease; T2D = type 2 diabetes; CVD = cardiovascular disease; AMI = acute myocardial infarction; ICM = ischaemic cardiomyopathy; MMPs = matrix metalloproteinases; PCSKs = proprotein convertase subtilisin/kexins; BMPs = bone morphogenetic proteins; CRP = C-reactive protein; VSMCs = vascular smooth muscle cells; MCP-1 = circulating monocyte chemotactic protein-1; VCAM-1 = vascular cell adhesion molecule 1; BMI = body mass index; MetS = metabolic syndrome; WC = waist circumference; LDL-C = low-density lipoprotein cholesterol; HDL-C = high-density lipoprotein cholesterol; SBP = systolic blood pressure; DBP = diastolic blood pressure; MAP = mean arterial pressure; CIMT = carotid intima-media thickness; MI = myocardial infarction; IHD = ischaemic heart disease; CV = cardiovascular; ↑ = increase; ↓ = decrease. * Directions of association reported by a majority of the human observational and genetic and animal studies included in this review, with the exception of a few studies that report opposite directions of association.

The evidence outlined above suggests that furin plays a complex role in several different pathways leading to the development of CVD, including inflammation, lipid regulation, and atherogenesis (Figure 2). The majority of the available evidence from human population studies indicates a positive relationship between furin and CVD development and progression. Several associations have been identified between CVD risk factors and outcomes and variants within the *FURIN* gene, with CAD and blood pressure being among the most reported phenotypes, and relatively limited evidence on other CVD outcomes such as stroke and heart failure, with few studies in diverse ancestry populations. However, the direction of association in genetic studies is not always clear, and further research is needed to disentangle the relationships between these genetic variants, furin expression patterns, protein levels, and CVD risk factors and outcomes. There also remains a question around the biological relevance of circulating furin levels on CVD risk, given that furin mainly localises to the trans-Golgi network within cells. Further investigations in cellular and animal models could help understand the relevance of circulating furin protein and its mechanisms of action in relation to CVD risk.

Fewer studies report on furin protein abundance in relation to CVD risk, and there are some notable contrasting results with regard to the direction of association with CVD risk factors and outcomes. It is also possible that furin levels are in turn affected by atherosclerosis progression and its biological consequences. However, the accumulating evidence indicating associations of higher levels of furin with higher levels of CVD risk and risk factors has prompted several reviews on its potential as a drug target for CVD [7,15,18,92,93,94,95]. Suur et al. recently scored furin as the fourth highest novel druggable target for CVD within the PCSK family following a series of detailed analyses, including protein–protein interaction, tissue expression, and clinical association analyses [95].

However, its ubiquitous expression and activity has led some researchers to emphasise exercising caution in pursuing it as a drug target [7,94], and the complications surrounding complete loss of function in all of the PCSK family have been outlined in detail [96]. Despite this, however, there are several inhibitors known to target furin activity and expression. These are a mixture of non-peptide small molecules, peptide-based or peptidomimetic (small protein-like chains that are similar to peptides) inhibitors [8], with two natural molecules and an anti-coagulant recently being added to the list [97]. Some of their differences in molecular structure, targets, and indications have been previously discussed [8,93]. Recently, they have become the subject of increased attention due to their potential as treatments for SARS-CoV-2 infection [8,98,99,100]. Yet evidence of their use in cardiovascular research is limited, and, as far as is known, none have progressed further than animal and cell models. To our knowledge, only one agent developed to target furin has progressed into clinical development programmes, involving mainly phase 1 and 2 clinical trials for cancer treatment [25,93]. The FANG vaccine (also known as Vigil) is an immunotherapy that uses bifunctional shRNA to inhibit furin activity [25]. During phase 2 trials, this vaccine improved survival in individuals with late-stage ovarian cancer [26,101] and Ewing sarcoma [102]. The rationale for the focus on these particular cancer types is unclear; however, both are typically only diagnosed at a late stage, with poor prognosis and high rates of recurrent disease, making them particularly difficult to treat successfully using current methods. Reported adverse events mostly occurred at the injection site and ranged from bruising, erythema, and induration to fatigue, pain, and swelling [102]. The lack of serious adverse events from these early trials is promising when considering drug purposing; however, these studies are still relatively small, with restricted follow-up, and are limited by participant numbers or early termination. Although larger advanced-stage trials could provide more information on the safety profile of this intervention, the results from these early-stage studies have provided sufficient support for further trials, and the latest randomised, double-blind phase 2 trial for advanced ovarian, fallopian tube, or primary peritoneal cancer is currently ongoing (NCT02346747, https://clinicaltrials.gov/ accessed on 15 January 2024). However, to our knowledge, there is no evidence of the efficacy of this technology for prevention or treatment of CVD, and there are no current clinical trials of pharmaceutical products targeting furin for CVD. Evidence from these cancer trials may inform the further development of furin-targeted treatment for CVD.

**Figure 3:**
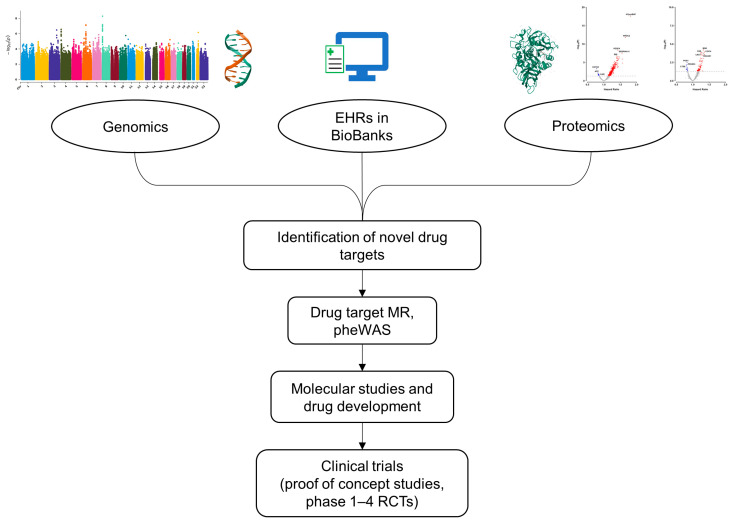
New approaches in proteo-genomics can inform drug development. Abbreviations: EHRs = electronic health records; MR = Mendelian randomisation; pheWAS = phenome-wide association studies; RCTs = randomised controlled trials. Furin protein structure downloaded from https://www.opentargets.org/ accessed on 15 January2024.

Other major challenges to research into furin as a drug target candidate are cost and time. Recent estimates suggest that the cost of developing and delivering a new drug to market is in the region of $1.6 billion per product [103] and can take upwards of 15 years [104]. However, findings from genetic studies can improve drug target approval rates. The likelihood of success of progressing from phase 1 trials to approval for drug targets with genetic support is more than double than those without [105]. Large-scale biobanks in diverse ancestry populations with electronic health records and genetic data on thousands of participants, together with recent advances in proteomics assays, afford new opportunities to inform drug target research [106] (Figure 3). Techniques such as Mendelian randomisation (MR), colocalisation, and phenome-wide association studies (pheWAS) can be used to investigate the effects of furin inhibition on a wide range of outcomes, although, given its ubiquitous expression and various functions, it may be challenging to disentangle different effects in specific tissues using these methods. With improvements in proteomics technologies, data from proteo-genomic studies are likely to become an increasingly important resource to inform drug development. The UKB Pharma Proteomics Project (UKB-PPP) provides pQTL mapping for nearly 2300 proteins measured on the Olink Explore 3072 platform in >54,000 participants, and larger sample sizes will improve the power and strength of genetic instruments for use in drug target MR analysis [107]. Further research is warranted into new furin-based treatments for CVD, repurposing the potential of existing technologies such as FANG/Vigil, or developing the known small molecule furin inhibitors, and will require effective interdisciplinary collaborations combining molecular biology, laboratory and population studies, and clinical research.

## Figures and Tables

**Table 1:** Summary of FURIN gene associations with cardiovascular risk factors.

Study Design	Study Population	SNP	Chr	Mapped Genes	Minor Allele	Associated Trait(s)	Reference
Blood pressure and hypertension:							
Case-control	Turkic N = 934	rs2071410	15	*FURIN*	A/G/T	Hypertension (G allele = ↑ risk)	[19]
Cross-sectional	European N = 1428	rs4702	15	*FURIN*	A/C	Protein expression in blood (A allele = ↓ levels), SBP and DBP (A allele = ↑ levels)	[20]
Intervention	Multiple ancestries N = 23	rs12917264	15	*FURIN*	T	Post-exercise hypotension **	[28]
		rs75493298	15	*FURIN*	A/T	Post-exercise hypotension **	
		rs74037507	15	*FURIN*	A/C	Post-exercise hypotension **	
		rs1573644	15	*FURIN*	C	Post-exercise hypotension **	
Cross-sectional	European N = 61,619	rs2071410	15	*FURIN*	A/G/T	DBP **	[29]
		rs6227	15	*FURIN*	T	SBP, MAP **	
Case-control, cross-sectional	Multiple ancestries N = 276,527	rs2521501	15	*FES*	C/T	SBP, DBP (T allele = ↑ levels)	[30]
Case-control, cross-sectional	European N = 69,395	rs8032315	15	*FURIN*	A/C	SBP	[31]
Cross-sectional	Multiple ancestries N = 130,828	rs6227	15	*FURIN*	T	SBP **	[32] *
		rs1573643	15	*FURIN*	C/G	DBP, MAP **	
		rs8027450	15	*FURIN*	A/T	PP **	
		rs8029440	15	*RN7SL363P, FURIN*	A	DBP **	
Cross-sectional	Multiple ancestries N = 178,726	rs7166599	15	*RN7SL363P, FURIN*	C/G	SBP, MAP (G allele = ↑ levels)	[33] *
		rs12906125	15	*FES—FURIN*	A	SBP (A allele = ↑ levels)	
		rs1573643	15	*FURIN*	C/G	MAP (C allele = ↑ levels)	
Longitudinal	Multiple ancestries N = 99,785	rs4932371	15	*RN7SL363P, FURIN*	C	SBP, DBP, PP (T allele = ↓ levels)	[34] *
Cross-sectional	Europeans N = 226,997	rs8032315	15	*FURIN*	A/C	SBP (A allele = ↑ levels)	[35] *
Cross-sectional	Europeans N = 396,077	rs8032315	15	*FURIN*	A/C	SBP (A allele = ↑ levels)	[36] *
Cross-sectional	Europeans N = 68,450	rs2071410	15	*FURIN*	A/G/T	DBP/depression **	[37] *
Case-control	Europeans N = 56,637	rs57515981	15	*FURIN*	AAAGGAAG, AAAGGCAG, AAGGCAG	Hypertension (AAAGGCAG alleles = ↑ risk)	[38] *
Cross-sectional, case-control	Multiple ancestries N = 146,562	rs17514846	15	*FURIN*	A/G/T	SBP, DBP, MAP (C allele = ↓ levels)	[39] *
Cross-sectional	East Asian N = 162,255	rs17514846	15	*FURIN*	A/G/T	SBP **	[40] *
Cross-sectional	East Asian N = 130,777	rs17514846	15	*FURIN*	A/G/T	SBP and MAP (A allele = ↑ levels) and hypertension (A allele = ↑ risk)	[41] *
		rs8039305	15	*FURIN*	C	Hypertension (T allele = ↓ risk)	
Case-control	Europeans N = 218,792	rs8032315	15	*FURIN*	A/C	Antihypertensive use **	[42] *
Case control	European N = 15,200	rs6224	15	*FURIN*	C/T	Preeclampsia or other hypertensive disorders during pregnancy (T allele = ↑ risk)	[43] *
Case-control	Multiple ancestry N = 468,391	rs6224	15	*FURIN*	C/T	Preeclampsia and gestational hypertension (T allele = ↑ risk)	[44]
Blood lipids:							
Cross-sectional	Multiple ancestries N = 178,726	rs8039305	15	*FURIN*	C	Taking lipid-lowering medication—HMG-CoA reductase inhibitors (C10AA; C allele = ↑ use)	[33] *
Case-control	East Asian N = 5460	rs17514846	15	*FURIN*	A/G/T	Serum triglycerides (A allele = ↓ levels)	[45]
Case-control	East Asian N = 2918	rs17514846	15	*FURIN*	A/G/T	MetS (A allele = ↓ risk), serum triglycerides (A allele = ↓ levels), and serum HDL-C (A allele = ↑ levels)	[46]

SNPs were characterised using the dbSNP (https://www.ncbi.nlm.nih.gov/snp/ accessed on 15 January 2024) and GWAS Catalog (https://www.ebi.ac.uk/gwas/ accessed on 15 January 2024) databases. Search strategy: studies were identified using PubMed (https://pubmed.ncbi.nlm.nih.gov/ accessed on 15 January 2024; search terms: “Furin, CVD”, “Furin, cardiovascular disease”) and the GWAS Catalog, and abstracts were screened manually for mention of any CVD-related outcomes or risk factors, including IHD, stroke, coronary artery disease (CAD), myocardial infarction (MI), blood pressure, hypertension, blood lipids, diabetes, and heart failure (HF). Abbreviations: DBP = diastolic blood pressure; SBP = systolic blood pressure; MAP = mean arterial pressure; ICBP = International Consortium for Blood Pressure; PP = pulse pressure; MetS = metabolic syndrome; HMG-CoA = hydroxymethylglutaryl coenzyme A; ↑ = increase; ↓ = decrease. * SNP associations identified from the GWAS Catalog. ** Effect allele unclear.

**Table 3:** Summary of FURIN gene associations with cardiovascular disease outcomes.

Study Design	Study Population	SNP	Chr	Mapped Genes	Minor Allele	Associated Trait(s)	Reference
Coronary artery disease:							
Two sample MR	Multiple ancestries N = >20,000	rs6227	15	*FURIN*	C/T	IHD **	[4]
		rs1029420	15	*FURIN*	C	IHD **	
Case-control	Multiple ancestries N = 194,427	rs17514846	15	*FURIN*	A/G/T	CAD (A allele = ↑ risk)	[23]
Case-control, cross-sectional	European N = 69,395	rs8032315	15	*FURIN*	A/C	CAD **	[31]
Cross-sectional	Multiple ancestries N = 178,726	rs57515981	15	*FURIN*	AAAGGAAG, AAAGGCAG, AAGGCAG	UAP (AAAGGCAG alleles = ↑ risk) and WBC (AAAGGCAG alleles = ↑ levels)	[33] *
Longitudinal	Multiple ancestries N = 99,785	rs4932371	15	*RN7SL363P, FURIN*	C	CAD (C allele = ↑ risk)	[34] *
Cross-sectional, case-control	Multiple ancestries N = 146,562	rs17514846	15	*FURIN*	A/G/T	CAD and MI (C allele = ↓ risk)	[39] *
Case-control	Europeans N = 218,792	rs8032315	15	*FURIN*	A/C	CAD	[42] *
Cross-sectional	European N = 826	rs17514846	15	*FURIN*	A/G/T	Circulating MCP-1 (A allele = ↑ levels), and CIMT (A allele = ↑ thickness)	[59]
Case-control	European N = 408,458	rs6227	15	*FURIN*	T	IHD **	[61]
		rs17514846	15	*FURIN*	A/G/T	IHD **	
Case-control	Multiple ancestries N = 184,305	rs17514846	15	*FURIN*	A/G/T	CAD (A allele = ↑ risk)	[74] *
Case-control	Multiple ancestries N = 738,986	rs8027450	15	*FURIN*	A/T	CAD with haemostatic traits **	[75] *
		rs8032315	15	*FURIN*	A/C	CAD, PAI-1, tPA **	
Case-control	Multiple ancestries N = 392,241	rs4932371	15	*RN7SL363P, FURIN*	C	CAD (C allele = ↑ risk)	[76] *
Case-control	Multiple ancestries N = 296,525	rs17514846	15	*FURIN*	A/G/T	CAD (A allele = ↑ risk)	[77] *
		rs4932373	15	*FES*	C/T	CAD (A allele = ↓ risk)	
Case-control	European N = 20,978	rs17514846	15	*FURIN*	A/G/T	Identified as critical component in several different CAD pathways	[78]
Stroke:							
Case-control	East Asian N = 753	rs2071410	15	*FURIN*	A/G/T	TIA (G allele = ↑ risk), 90-day prognosis (G allele = ↓ prognosis)	[79]
Case-control	Multiple ancestries N = 521,612	rs4932370	15	*RN7SL363P, FURIN*	A/C/T	AIS **	[80] *
Case-control	Multiple ancestries N = 1,614,080	rs1573644	15	*FURIN*	C	LAS, AS and AIS (C allele = ↑ risk)	[81]

SNPs were characterised using the dbSNP (https://www.ncbi.nlm.nih.gov/snp/ accessed on 15 January 2024) and GWAS Catalog (https://www.ebi.ac.uk/gwas/ accessed on 15 January 2024) databases. Search strategy: studies were identified using PubMed (https://pubmed.ncbi.nlm.nih.gov/ accessed on 15 January 2024; search terms: “Furin, CVD”, “Furin, cardiovascular disease”) and the GWAS Catalog, and abstracts were screened manually for mention of any CVD-related outcomes or risk factors, including IHD, stroke, coronary artery disease (CAD), myocardial infarction (MI), blood pressure, hypertension, blood lipids, diabetes, and heart failure (HF). Abbreviations: MR = Mendelian Randomisation; CAD = coronary artery disease; MI = myocardial infarction; CC4D = Coronary ARtery DIsease Genome wide Replication and Meta-analysis (CARDIoGRAM) plus The Coronary Artery Disease (C4D) Genetics consortium; PAI-1 = plasminogen activator inhibitor-1; tPA = tissue plasminogen activator; UAP = unstable angina pectoris; WBC = white blood cell count; IHD = ischaemic heart disease; MCP-1 = monocyte chemotactic protein-1; CIMT = carotid intima-media thickness; TIA = transient ischaemic attack; AIS = any ischaemic stroke; LAS = large-artery atherosclerotic stroke; AS = any stroke; ↓ = decrease; ↑ = increase. * SNP associations identified from the GWAS Catalog. ** Effect allele unclear.

**Table 4:** Summary of associations between furin protein levels or activity and cardiovascular disease outcomes.

Study Design	Population	Primary Endpoints	Protein Measurement	Key Findings	Reference
Coronary artery disease					
Case-subcohort (China)	Incident CAD cases and subcohort from CKB prospective cohort study (N = 3977)	CAD	Plasma furin levels measured using Olink Explore 1500 assay	Higher risk of CAD associated with higher plasma furin levels	[4]
Case control (Shanghai, China)	STEMI and non-STEMI patients admitted to Tongji Hospital and Shanghai East Hospital, Tongji University (N = 1312)	MACE (including all-cause mortality, hospitalisation for HF and recurrent MI)	Furin serum levels measured using ELISA	Risk of MACE and all-cause mortality significantly higher for those in the highest furin concentration Risk of recurrent MI, cardiovascular death, and rehospitalisation for HF were also significantly higher for those with the highest furin concentrations Furin significantly improved prediction models	[50]
Prospective, (Suita, Japan)	City residents aged 30–79 years (N = 1436)	CAD (including AMI, sudden cardiac death, and stable CAD)	Total, mature, and fc-PCSK9 measured using ELISA	Highest tertile of fc-PCSK9 was associated with a higher risk of coronary and composite events compared to the lowest tertile Fc-PCSK9 predicted future coronary events whereas the mature subtype did not demonstrate any association with CV outcomes	[53]
Prospective, (Kanazawa, Japan)	Hospitalised STEMI patients (N = 126)	MACE (composite of all-cause mortality, non-fatal MI and stroke, and new angina pectoris)	Mature and fc-PCSK9 measured using ELISA	Patients in the highest tertile of fc/m-PCSK9 serum levels were at significantly higher risk of MACE during the follow-up period	[65]
Prospective (Beijing, China)	Hospitalised AMI patients admitted to the People’s Liberation Army General Hospital (N = 1100)	MACE (composite of CV death, non-fatal MI, non-fatal stroke)	Furin serum levels measured using ELISA	Significant association between elevated furin levels at baseline and recurrent non-fatal MI, but not with MACE or CV death	[82]
Other cardiovascular diseases					
Cross-sectional (Monastir, Tunisia; Paris, France; Finland)	Emergency room patients presenting with shortness of breath (“Biomarcoeurs” cohort) and AHF patients from 14 hospitals (FINN-AKVA cohort) (N = 683)	ADHF and CHF	Furin serum levels measured using ELISA, furin activity measured using fluorescence	No significant difference in furin plasma concentrations of between groups Furin activity was significantly higher in ADHF cases No correlation between furin concentration and plasma levels of proBNP, NT-proBNP, and BNP	[83]
Case-control (Valencia, Spain)	Patients at “La Fe” University Hospital (N = 73)	DCM and ICM	Furin tissue protein levels of measured by gel electrophoresis and western blot	No significant difference in furin protein levels between HF patients and controls Furin levels were significantly higher in ICM patients compared to DCM patients	[84]

Search strategy: studies were identified using PubMed (https://pubmed.ncbi.nlm.nih.gov/ accessed on 15 January 2024; search terms: “Furin, CVD”, “Furin, cardiovascular disease”), and abstracts were screened manually for mention of any CVD-related outcomes or risk factors, including IHD, stroke, coronary artery disease (CAD), myocardial infarction (MI), blood pressure, hypertension, blood lipids, diabetes, and heart failure (HF). Abbreviations: CKB = China Kadoorie Biobank; AHF = acute heart failure; ADHF = acute decompensated heart failure; CHF = congestive heart failure; ELISA = enzyme-linked immunosorbent assay; NT-proBNP = N-terminal prohormone of brain natriuretic peptide; BNP = brain natriuretic peptide; DCM = dilated cardiomyopathy; ICM = ischaemic cardiomyopathy; HF = heart failure; CAD = coronary artery disease; STEMI = ST segment elevation myocardial infarction; non-STEMI = non-ST segment elevation myocardial infarction; MACE = major adverse coronary event; MI = myocardial infarction; AMI = acute myocardial infarction; CV = cardiovascular; PCSK9 = proprotein convertase subtilisin/kexin type 9.

## Data Availability

No new data were created or analysed in this study.

## Abbreviations

α-1-PDX	α1-antitrypsin Portland
ADHF	Acute decompensated heart failure
AMI	Acute myocardial infarction
BBJ	Biobank Japan
bi-shRNA	Bifunctional small hairpin RNA
BMI	Body mass index
BNP	Brain natriuretic peptide/B-type natriuretic peptide
CAD	Coronary artery disease
CC4D	CARDIoGRAM*plus*C4D
CHF	Congestive heart failure
CIMT	Carotid intima media thickness
CKB	China Kadoorie Biobank
CRP	C-reactive protein
CVD	Cardiovascular disease
DALYs	Disability adjusted life years
DBP	Diastolic blood pressure
DCM	Dilated cardiomyopathy
ENaC	Epithelial sodium channel
eQTL	Expression quantitative trait loci
Fc-PCSK9	Furin-cleaved PCSK9
FDR	False discovery rate
GBD	Global Burden of Disease study
GEO	Gene Expression Omnibus
GTEx	Genotype Tissue Expression database
GWAS	Genome-wide association studies
HDL-C	High-density lipoprotein cholesterol
HeFH	Heterozygous familial hypercholesterolemia
HF	Heart failure
HR	Hazard ratio
ICBP	International consortium for blood pressure
ICM	Ischaemic cardiomyopathy
ICU	Intensive care units
IHD	Ischaemic heart disease
IL1-β	Interleukin-1 beta
LAS	Large-artery atherosclerotic stroke
LDL-C	Low-density lipoprotein cholesterol
LDLR	Low-density lipoprotein lipase receptors
MACE	Major adverse coronary events
MAP	Mean arterial pressure
MCAO	Middle cerebral artery occlusion
MCP-1	Monocyte chemotactic protein-1
MetS	Metabolic syndrome
MeCP2	Methyl-CpG binding protein 2
MI	Myocardial infarction
MiRNA	MicroRNA
mRNA	Messenger RNA
MMPs	Matrix metalloproteinases
MR	Mendelian randomisation
NSTEMI	Non-ST segment elevation myocardial infarction
OR	Odds ratio
PCSK6	Proprotein convertase subtilisin/kexin type 6
PCSK9	Proprotein convertase subtilisin/kexin type 9
PCR	Polymerase chain reaction
PheWAS	Phenome-wide association studies
PLTP	Phospholipid transfer protein
pQTL	Protein quantitative trait loci
PRR	Pro-renin receptor
SBP	Systolic blood pressure
SD	Standard deviation
shRNA	Short-hairpin RNA
SNP	Single nucleotide polymorphism
STEMI	ST-elevation myocardial infarction
T2D	Type 2 diabetes
TAA	Thoracic aortic aneurysms
TGF-β1	Transforming growth factor beta 1
TIA	Transient ischaemic attack
TNF-α	Tumour necrosis factor alpha
UKB	UK Biobank
VCAM-1	Vascular cell adhesion molecule-1
